# A proposed tree-based explainable artificial intelligence approach for the prediction of angina pectoris

**DOI:** 10.1038/s41598-023-49673-2

**Published:** 2023-12-14

**Authors:** Emek Guldogan, Fatma Hilal Yagin, Abdulvahap Pinar, Cemil Colak, Seifedine Kadry, Jungeun Kim

**Affiliations:** 1 Department of Biostatistics and Medical Informatics, Faculty of Medicine, Inonu University, 44280 Malatya, Turkey; 2 Noroff University College, Kristiansand, Norway; 3 Artificial Intelligence Research Center (AIRC), Ajman University, 346 Ajman, United Arab Emirates; 4 Department of Electrical and Computer Engineering, Lebanese American University, Byblos, Lebanon; 5 Department of Software, Kongju National University, Cheonan, 31080 Korea

**Keywords:** Diseases, Medical research, Risk factors

## Abstract

Cardiovascular diseases (CVDs) are a serious public health issue that affects and is responsible for numerous fatalities and impairments. Ischemic heart disease (IHD) is one of the most prevalent and deadliest types of CVDs and is responsible for 45% of all CVD-related fatalities. IHD occurs when the blood supply to the heart is reduced due to narrowed or blocked arteries, which causes angina pectoris (AP) chest pain. AP is a common symptom of IHD and can indicate a higher risk of heart attack or sudden cardiac death. Therefore, it is important to diagnose and treat AP promptly and effectively. To forecast AP in women, we constructed a novel artificial intelligence (AI) method employing the tree-based algorithm known as an Explainable Boosting Machine (EBM). EBM is a machine learning (ML) technique that combines the interpretability of linear models with the flexibility and accuracy of gradient boosting. We applied EBM to a dataset of 200 female patients, 100 with AP and 100 without AP, and extracted the most relevant features for AP prediction. We then evaluated the performance of EBM against other AI methods, such as Logistic Regression (LR), Categorical Boosting (CatBoost), eXtreme Gradient Boosting (XGBoost), Adaptive Boosting (AdaBoost), and Light Gradient Boosting Machine (LightGBM). We found that EBM was the most accurate and well-balanced technique for forecasting AP, with accuracy (0.925) and Youden's index (0.960). We also looked at the global and local explanations provided by EBM to better understand how each feature affected the prediction and how each patient was classified. Our research showed that EBM is a useful AI method for predicting AP in women and identifying the risk factors related to it. This can help clinicians to provide personalized and evidence-based care for female patients with AP.

## Introduction

Cardiovascular diseases (CVDs) indeed pose a significant global health burden and have become a major cause of mortality and morbidity worldwide. CVDs include a range of conditions affecting the heart and blood vessels, such as coronary artery disease, heart attacks, stroke, heart failure, and arrhythmias^1^. Prevention and management strategies are crucial in reducing the burden of CVDs. Lifestyle modifications, such as adopting a healthy diet, engaging in regular physical activity, avoiding tobacco use, and managing stress, can significantly lower the risk of developing CVDs. Early detection, timely medical intervention, and access to quality healthcare services are essential for effectively managing CVDs and preventing complications^2,3^. Ischemic heart disease (IHD) is indeed a major cause of death among CVDs^4^. IHD refers to the condition where the myocardium (heart muscle) does not receive enough blood and oxygen, leading to various clinical manifestations. The primary cause of IHD is the narrowing of the coronary arteries due to the formation of atherosclerotic plaques, which restrict blood flow to the heart. This reduced blood flow creates an imbalance between the demand for oxygen by the myocardium and the actual supply of oxygen^4,5^. One of the most prominent symptoms of IHD is angina pectoris (AP). AP is characterized by discomfort or pain in the chest, arm, shoulder, back, or jaw. It occurs when there is an increased demand for oxygen at the cellular level in the heart or a decrease in the oxygen concentration within the myocardium. While the narrowing of coronary arteries is commonly associated with decreased oxygen supply, other factors such as increased heart rate, untreated hypertension (high blood pressure), and heightened myocardial contractility can also contribute to AP^6–8^. Women may have less obstructive coronary artery disease and better left ventricular function than men. Still, they experience higher morbidity, mortality, and worse quality-of-life outcomes when they develop AP. Even without significant obstructive coronary artery disease, women who experience chest discomfort and myocardial ischemia (reduced blood supply to the heart muscle) are at substantial risk of death and morbidity^9–11^.

Machine learning (ML) is one of the most appropriate techniques for developing models used in the healthcare industry, particularly for diagnosing diseases. ML is a field of artificial intelligence that focuses on developing models or systems capable of learning from existing data sets and making predictions or taking actions based on that learning. In healthcare, ML algorithms are used to analyze large amounts of data, uncover meaningful patterns, and extract valuable insights. By applying ML algorithms to healthcare data, valuable insights can be obtained to enhance diagnostic decision-making. ML models can learn from large datasets of diagnostic data, identify important patterns during the learning process, and reduce the need for human intervention in decision-making. This can lead to more accurate and efficient diagnoses, improved patient outcomes, and reduced healthcare costs^12–14^. A recent study, established a prediction model for the occurrence of angina pectoris events using Bi-directional Long Short-Term Memory (Bi-LSTM) with the attention layer to explore the predictive value of the resting-state RR interval time series on the occurrence of AP, and the model achieved good prediction performance with an accuracy of 91.3%^15^.

Explainable Boosting Machine (EBM), Categorical Boosting (CatBoost), eXtreme Gradient Boosting (XGBoost), Adaptive Boosting (AdaBoost), Light Gradient Boosting Machine (LightGBM), and Logistic Regression (LR) are powerful ML algorithms that have been successfully applied in disease detection and other medical applications^16–18^.

This study aims to investigate biomarker candidate risk factors for detecting, monitoring, and treating AP in women and to compare the prediction performances of tree-based ML models for AP prediction based on these risk factors.

The main contributions of this paper include the creation of a new dataset for AP disease, the comparison of different ML algorithms to predict AP, and the development of an explainable approach belongs to the family of GAMs—Generalized Additive Models, to accurately predict the AP events with the interpretation of the results. To our knowledge, this is the first study using EBM to predict AP events from an explainable AI/ML (XAI/XML) perspective.

## Material and methods

### Dataset, related factors, and ethics approval

The purpose of this research dataset was to investigate the possibility of predicting AP in females and to identify the risk factors that are associated with this condition. The public (openly accessible) dataset included 200 female patients who were examined for the presence or absence of AP, as well as for several other variables that could potentially influence the development of AP. The open access dataset used in the study was obtained from the web address (https://www.kaggle.com/datasets/snehal1409/predict-angina). The patients were divided into two groups: 100 patients (50%) who had been diagnosed with AP and 100 patients (50%) who did not have AP. The variables that were examined for each patient were: smoking habits (whether they smoked or not), age (in years), family history of angina (whether any of their first-degree relatives had angina), hypertension status (whether they had high blood pressure or not), amount of cigarettes consumed per day (in number), family history of myocardial infarction (whether any of their first-degree relatives had a heart attack), and family history of stroke and diabetes (whether any of their first-degree relatives had a stroke or diabetes). These variables were carefully analyzed to determine their possible roles in predicting the occurrence of angina pectoris in the female population under study. Table 1 shows the input and output factors/features that were used in the analysis^19^. This study was conducted per the principles of the Declaration of Helsinki and was approved by the Inonu University Non-invasive Clinical Research Ethics Committee (decision no: 2023/4976). Informed consent was obtained from all subjects participating in the related study.

**Table 1:** The input and output factors/features under question.

Variable/feature	Description	Type	Role
Status	Whether a woman turns out to have angina pectoris (0 = no, 1 = yes)	Categorical	Output
Age	Age of a woman	Continuous	Input
Smoke	Smoking status (1 = current-, 2 = ex-, 3 = non-smoker)	Categorical	Input
Cigarette	The current average number of cigarettes per day	Continuous	Input
Hyper	Hypertension (1 = absent, 2 = mild, 3 = moderate)	Categorical	Input
Angfam	Family history of angina (1 = yes.0 = no)	Categorical	Input
Myofam	Family history of myocardial infarction (1 = yes, 0 = no)	Categorical	Input
Strokefam	Family history of stroke (1 = yes, 0 = no)	Categorical	Input
Diabetes	Does a woman have diabetes mellitus? (1 = yes, 0 = no)	Categorical	Input

### Biostatistical data analysis

Qualitative variables were summarized by calculating frequency (percentage). Pearson chi-square test, Yates continuity correction test, and Fisher’s exact test were employed to examine the relationships of the qualitative variables with AP where appropriate. When any of the categories of qualitative variables is > 2, the Pearson chi-square test with Bonferroni adjustment was preferred where appropriate instead of other techniques^20^. In multivariate analysis, possible risk factors for AP were examined by binary logistic regression analysis. Hosmer–Lemeshow and Omnibus tests were used to evaluate the logistic regression model and its coefficients^21^. In all results, *p* ≤ 0.05 was considered statistically significant. Statistical analyses were performed using the SPSS 28.0 (IBM Corp., Armonk, NY, United States) package program. The calculated (post-hoc) power (1-beta) based on the most important factor from the EBM model result was nearly 1, considering type I error (alpha) of 0.05, sample size of 100 in each group (200 in total), effect size of 1.36 and two-sided alternative hypothesis (H1)^22^.

### ML approaches

Gradient boosting, a ML technique, is utilized for classification problems by producing a strong overall prediction through an ensemble of multiple weak models. Typically employing decision trees, the ensemble method uses a weighted average to combine these models' predictions, where trees performing better on training data are assigned higher weights. With each iteration of boosting, weights are updated to focus more on previously misclassified samples, culminating in an ensemble prediction based on a weighted majority vote. For evaluating models predicting acute pancreatitis (AP) patients, a validation method of 5-times repeated tenfold cross-validation (CV) was employed. CV, a technique to gauge an ML model's generalizability to unseen data, involves dividing the dataset into folds, training the model on a subset, and evaluating it on the rest. In tenfold CV, the dataset is split into ten equal parts, with each part used once for evaluation and the rest for training, repeated ten times. This procedure, repeated 5 times with new random partitions, offers a robust assessment of the model's performance and reduces variability in performance estimates^23–25^.

The model's effectiveness was gauged using various metrics, including Accuracy, F1-Score, Sensitivity, Specificity, Youden's index, Positive Predictive Value (PPV), Negative Predictive Value (NPV), and Area Under the Curve (AUC). Additionally, a calibration curve based on isotonic regression was employed to ensure the model's predictions aligned well with actual results^26^. Isotonic regression, a model calibration technique in ML, adjusts predicted probabilities to match observed outcomes, enhancing the reliability and accuracy of these predictions in classification tasks. This calibration is vital for decision-making processes that depend on precise probability estimates^27,28^. Finally, to intuitively interpret the optimal model, both global and local annotations were created, providing a comprehensive understanding of the model's functioning and decision-making process.

#### Categorical boosting (CatBoost)

CatBoost is one of the ML methods that can work with categorical and numerical data. One distinctive feature of CatBoost is its capacity to alleviate overfitting by addressing noise points. This is achieved by introducing prior values at locations characterized by low-frequency features and high density. The technique was developed based on gradient-supported decision trees (GBDT). To appropriately understand data and assess conclusions, CatBoost overcomes the bias of the gradient-best descent approach and the drift of prediction values^29,30^. Both CPU and GPU versions of CatBoost exist. On ensembles of comparable sizes, the GPU implementation outperforms both cutting-edge open-source GBDT GPU implementations, XGBoost and LightGBM, and enables substantially quicker training. Additionally, the library offers a quick CPU scoring implementation that outperforms XGBoost and LightGBM on ensembles of comparable size. Notably, CatBoost excels in managing categorical features without preprocessing, directly substituting original categorical variables with numerical values. In models with overfitting issues, noise points are minimized by inserting a prior value at the points with low-frequency features and high density. This improves the model's generalization while minimizing the fit^31,32^. The CatBoost method can manage category features. The primary portion of this being processed, which is frequently done during the preprocessing stage, is to replace the original categorical variables with one or more numerical values. Additionally, it was found that CatBoost was capable of being successfully applied to a variety of data types and formats^33^. The approach's use of random permutations to estimate leaf values while choosing the tree structure, as noted, avoids the overfitting caused by traditional gradient techniques^31^.

#### Adaptive boosting (AdaBoost)

Freund and Schapire developed the AdaBoost method to integrate various algorithms into a robust, singular model^34^. This technique involves merging the output classes from different models, utilizing a training dataset to construct a diverse range of models^35^. As a renowned ensemble learning algorithm, AdaBoost enhances classification accuracy by adaptively reweighting and combining independent models. The method involves averaging negative and positive samples for each feature to determine the decision thresholds of weak classifiers^36^. Subsequently, AdaBoost selects the least error-prone weak classifiers for further refinement into stronger classifiers, discarding the attributes of weak classifiers that are not incorporated into the strong classifier^37^. AdaBoost also generates a series of hypotheses, focusing subsequent hypotheses on instances increasingly difficult to categorize. The final decision is based on the weighted majority vote of the classes predicted by all hypotheses. This systematic approach makes AdaBoost an effective tool for improving the precision of classification methods^34^.

#### Extreme gradient boosting (XGBoost)

Chen and Guestrin developed the XGBoost algorithm, an advanced gradient growth method, drawing parallels with GB decision trees and machines^38^. XGBoost is known for its efficiency in building parallel trees, offering rapid and precise models suitable for various engineering simulations. Its distinct feature, the "regular acceleration" technique, sets it apart from the typical Gradient Boosting models, which often omit this regularization step. To enhance accuracy, XGBoost integrates Gradient Boosting with innovative approaches, effectively combining multiple weak learners to bolster the overall learning effect^39,40^. The XGBoost architecture stands out for its strong flexibility and scalability, making it superior to traditional machine learning methods in boosting model performance. Frequently used in supervised learning, XGBoost is particularly effective in regression and classification tasks. Data scientists often prefer XGBoost for its quick outcomes, especially when calculating kernel functions^38^. The algorithm optimizes the learning process in complex structures by exploiting the objective function's standard normality, thereby accelerating the learning phase. This multifaceted approach contributes to XGBoost's widespread adoption in the field of data science^41^.

#### Light gradient boosting machine (LightGBM)

LightGBM, a computational methodology developed by Microsoft in 2016, stands out in the machine learning domain, especially among decision tree-based algorithms. Its most notable feature is the accelerated model training speed, primarily due to its innovative leaf-wise growth strategy for data training. This approach diverges from the traditional depth-wise or level-wise strategies found in other gradient boosting frameworks^42–44^. By utilizing the Gradient one-way sampling technique, LightGBM efficiently reduces data volume, focusing on relevant dataset sections instead of the entire data pool. LightGBM offers several advantages over other boosting methods. These include rapid processing, the capacity to handle large data volumes, reduced RAM usage, and enhanced prediction accuracy. Additionally, it supports parallel and GPU learning, making it a versatile and resource-efficient option^45,46^. As an open-source system, LightGBM builds upon the highly effective gradient boosting decision tree (GBDT) technology, showcasing Microsoft's contribution to advancing machine learning tools^47^.

#### Explainable boosting machine (EBM)

EBM, a tree-based cyclic gradient-boosting Generalized Additive Model (GAM) with automatic interaction detection, is a glass box model. It boasts accuracy comparable to advanced machine learning methods like Random Forest and Boosted Trees and excels in intelligibility and explainability. Unlike traditional models requiring simple weighted sums, GAMs interpret the outcome as the sum of arbitrary functions for each feature, enhancing interpretability. EBMs stand out due to their ability to identify and leverage unique trait combinations (interactions), boasting a compact size and rapid forecasting capabilities. In boosting, a group learning technique, weak learners are transformed into strong ones, optimizing performance. In EBM, the number of leaf nodes can be adjusted for further performance tuning. The boosting algorithm in EBM is meticulously crafted to focus on each feature independently during training iterations, with a feature-wise boosting approach. This strategy allows for low learning rates, making the order of feature consideration irrelevant to the final model. A significant challenge in model training is feature collinearity, which can hinder performance and interpretability. EBM addresses this by using numerous iterations in its training phase, enabling precise determination of each feature's contribution to the predictive output. Additionally, EBM can automatically detect and include pairwise interaction terms, enhancing predictive accuracy while maintaining explainability. This feature contrasts with traditional models that often require manual interaction term specification, which can complicate the model and obscure interpretability. The additive nature of EBM further contributes to its explainability by delineating each feature's individual impact on predictions, a stark contrast to the often opaque nature of more complex, black-box models. In summary, EBM not only retains the benefits of traditional GAMs but also improves upon them by offering enhanced accuracy, robustness, and in certain cases, superior explainability^48,49^.

**Figure 1:**
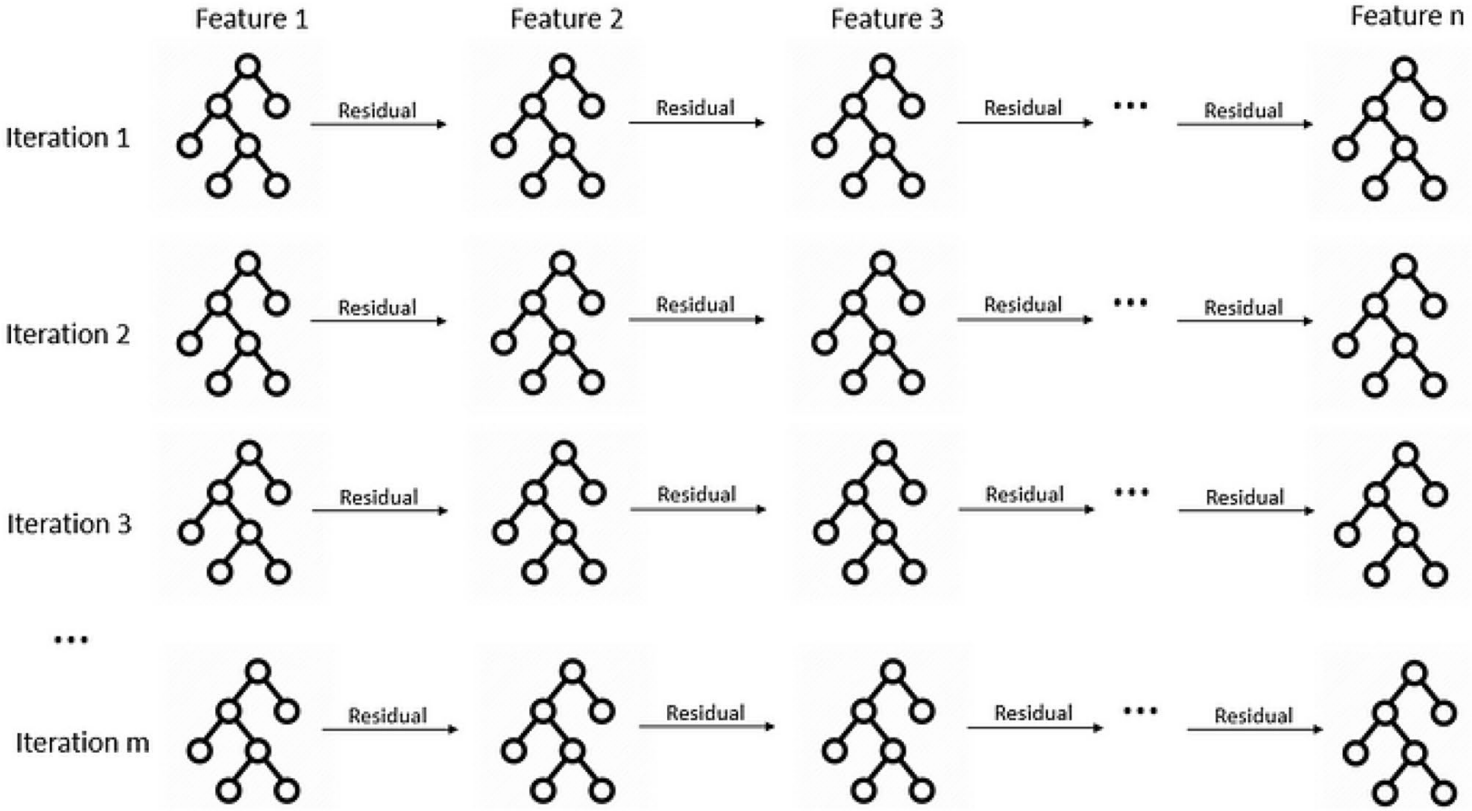
EBM algorithm.

The model constructs small trees sequentially for each iteration (Fig. 1), and each tree can only use one feature at a time. Boosting involves updating the residual and building a new tree based on a different feature. This is done for each feature in every iteration. Upon completion of training, we can build a graph showing the contribution of each feature to each tree constructed by a given feature^50^.

There are two types of explanations: global and local. The entire model and its general operation must be explained to provide a comprehensive understanding. Instead, a local explanation focuses on describing the result of a specific occurrence. A feature significance vector, or collection of values that reports a numeric value for each feature that is an input to an AI model and indicates how important that item is to the model's output, might serve as a local explanation. As one might imagine, an XAI model's outputs present numerous challenges for lay users^49,51^.

### Explaining and calibrating the optimum model

A model is deemed calibrated when its calculated probability matches the actual occurrence of outcomes^52^. For instance, if a model predicts a 0.9 risk of AP, it should correctly diagnose AP in 90% of such cases. This is vital for clinical decision-making, as it's important to know the model's confidence level in its predictions^53^. To ensure the estimated probabilities reflect true probabilities, the best model for AP estimation is calibrated. Isotonic regression is the preferred method for this calibration due to its generality. Unlike linear regression, isotonic regression is restricted to being monotonic (monotonically increasing) rather than linear, providing a more accurate adjustment for the model's predictions^54,55^. This calibrated model, optimized for both individual and cohort levels, is then applied, offering an intuitive and auditable approach in XAI. The use of isotonic regression in this context not only enhances the model's precision but also maintains its interpretability, crucial for practical applications in clinical settings^50^.

## Results

Descriptive statistics for qualitative factors are given in Table 2. 61 (74.4%) smokers and 26 (64.3%) ex-smokers had AP (*p* < 0.001). AP was observed in 53 (81.5%) of those with a family history of myocardial infarction (*p* < 0.001). Of those diagnosed with diabetes, 94 (49.2%) were AP (*p* < 0.001).

**Table 2:** Descriptive statistics on qualitative factors.

Variable	Categories	Group		*p*-value
		Control	AP	
		n (%)	n (%)	
Smoke	Current	21 (25.6)	61 (74.4)	< 0.001*
	Ex	15 (36.6)	26 (63.4)	
	Non-smoker	64 (83.1)	13 (16.9)	
Hypertension	Absent	83 (55.3)	67 (44.7)	0.22*
	Mild	14 (37.8)	23 (62.2)	
	Moderate	3 (23.1)	10 (76.9)	
Family history of angina	No	94 (52.5)	85 (47.5)	0.065**
	Yes	6 (28.6)	15 (71.4)	
Family history of myocardial infarction	No	88 (65.2)	47 (34.8)	< 0.001*
	Yes	12 (18.5)	53 (81.5)	
Family history of stroke	No	94 (50.0)	94 (50.0)	1**
	Yes	6 (50.0)	6 (50.0)	
Diabetes mellitus	No	1 (50.0)	1 (50.0)	< 0.001***
	Yes	97 (50.8)	94 (49.2)	

*Pearson Chi-square test, **continuity correction test, ***Fisher’s exact test.

Multivariate logistic regression analysis with backward feature selection and tree-based ML models (EBM, CatBoost, AdaBoost, XGBoost, and LightGBM) were applied to predict AP and identify important risk factors. The results of the multivariate logistic regression model for the detection of risk factors affecting AP are presented in Table 3. When Table 3 is examined, In Table 3, the coefficients for the independent variables, standard deviation, z-statistics, *p*-value, OR, 95% confidence level (CI) for OR, and the effect of independent variables on AP (reducing or increasing effect) and at the same time, effect size (ES) results indicating the severity/magnitude of this effect are given. The model result was not insignificant when the independent variables were analyzed with the Hosmer–Lemeshow test (*p* > 0.05). If the Hosmer–Lemeshow test is not significant, it indicates that the model has an acceptable fit and that the model-data fit is sufficient. The results of the test statistic of goodness of fit and the overall significance of the model coefficients (Omnibus test) were analyzed. According to the omnibus test results, the coefficients of the variables in the model are significant overall (*p* < 0.001). The results show that one-unit increase in age (OR = 1.125, 95% CI = [1.071–1.19], *p* < 0.001) and the mean amount of cigarettes consumed per day (OR = 1.078, 95% CI = [1.031–1.133], *p* = 0.002) showed that it increased the risk of AP 1.125 and 1.078-fold, respectively.

**Table 3:** Multiple logistic regression analysis results for predicting AP.

Variable	Coef	SD	z-statistics	*p*-value	OR	CI	Interpretation	ES
Constant	−7.384	1.522	−4.851	< 0.001	–	–	–	–
Age	0.118	0.027	4.412	< 0.001	1.125	1.071–1.19	Increasing effect	Small
Smoke_no	−1.952	0.555	−3.517	< 0.001	0.142	0.045–0.407	Reducing effect	Small
Amount of cigarettes consumed per day	0.075	0.024	3.128	0.002	1.078	1.031–1.133	Increasing effect	Small
Hypertension_mild	1.388	0.557	2.492	0.013	4.008	1.378–12.416	Increasing effect	Large
Hypertension_medium	2.239	0.915	2.448	0.014	9.38	1.685–64.42	Increasing effect	Large
Family history of angina_yes	1.446	0.697	2.076	0.038	4.246	1.139–17.891	Increasing effect	Large
Family history of myocardial infarction_yes	2.418	0.51	4.746	< 0.001	11.226	4.342–32.469	Increasing effect	Large

Hosmer–Lemeshow test			The goodness of fit statistics			Omnibus test		
Chi-squared	df	*p*-value	Chi-squared	df	*p*-value	Chi-squared	df	*p*-value
6.272	8	0.617	0.486	0.491	0.654	134.866	7	< 0.001

*Coef* coefficient, *SD* standard deviation, *OR* odds ratio, *CI* confidence interval, *ES* effect size, *df* degrees of freedom.

Non-smoking status, mild hypertension, mode LPBoost rate hypertension, family history of AP, and family history of myocardial infarction were also significantly included in the model. The risk of AP in non-smokers was 7.04-fold lower than in smokers (OR = 0.142, 95%CI = 0.045–0.407, *p* < 0.001). A family history of myocardial infarction increases the risk of AP 11.226 times compared to its absence (OR = 11.226, 95%CI = 4.342—32.469, *p* < 0.001). In addition, having a family history of myocardial infarction had the highest OR for AP. A family history of AP increased the risk of developing the disease 4.246-fold (OR = 4.246, 95%CI = 1.139–17.891, *p* = 0.038). Mild hypertension (OR = 4.008, 95%CI = 1.378–12.416, *p* = 0.013) and moderate hypertension (OR = 9.38, 95%CI = 1.685–64.42, *p* = 0.014) also increased the risk of AP by 4.008, and 9.38-fold, respectively.

The performance of an EBM model in AP classification was compared with CatBoost, AdaBoost, XGBoost, LightGBM, and LR models. The performance of all prediction models was comparable according to the results of accuracy, F1 score, sensitivity, specificity, Youden index, PPV, NPV, and AUC. Among the six ML classifiers, EBM performed best, with fast computation and strong generalization ability; therefore, the EBM model was used for AP prediction.

Moreover, the EBM model achieved very high sensitivity [0.955 (0.926–0.984)], specificity [0.950 (0.888–0.984)], and AUC [0.974 (0.952–0.995)]. A higher sensitivity value means a lower false negative (FN) value. False positive and false negative errors are common in comparative biological research. Therefore, it is crucial to determine the likelihood that a true effect will be significant. A lower FN value is an encouraging result for AP cases. This result is very important because minimizing missed AP cases (false negatives) is one of the main goals of this research.

The CatBoost model achieved an accuracy of 0.945 (0.931–0.977) and an F1 score of 0.945 (0.913–0.976) in predicting AP. When the results of the CatBoost model are compared with the other four prediction models (AdaBoost, XGBoost, LightGBM, and LR), it is seen that higher performance is achieved. The sensitivity, and specificity of the CatBoost model were 0.949 (0.886–0.983), and 0.941 (0.875–0.978). Black box models such as CatBoost can achieve performance benefits, but come with loss of interpretability and potentially much higher computing requirements. Based on this, EBM was used as the optimal model in AP prediction due to both the performance results and the absence of a black box (Table 4).

**Table 4:** Results of performance measures for AP prediction of the models.

Model	Accuracy	F1-score	Sensitivity	Specificity	Youden's index	PPV	NPV	AUC
EBM	0.955 (0.926–0.984)	0.955 (0.926–0.984)	0.950 (0.888–0.984)	0.960 (0.900–0.989)	0.910 (0.788–0.973)	0.960 (0.901–0.989)	0.950 (0.887–0.984)	0.974 (0.952–0.995)
CatBoost	0.945 (0.913–0.977)	0.945 (0.913–0.976)	0.949 (0.886–0.983)	0.941 (0.875–0.978)	0.890 (0.761–0.961)	0.940 (0.874–0.978)	0.950 (0.887–0.984)	0.969 (0.945–0.993)
AdaBoost	0.850 (0.801–0.899)	0.842 (0.792–0.893)	0.889 (0.805–0.945)	0.818 (0.733–0.885)	0.707 (0.538–0.831)	0.800 (0.708–0.873)	0.900 (0.824–0.951)	0.906 (0.853–0.959)
XGBoost	0.885 (0.841–0.929)	0.881 (0.836–0.926)	0.914 (0.838–0.962)	0.860 (0.779–0.919)	0.774 (0.617–0.881)	0.850 (0.765–0.914)	0.920 (0.848–0.965)	0.931 (0.887–0.975)
LightGBM	0.905 (0.864–0.946)	0.903 (0.861–0.944)	0.926 (0.854–0.97)	0.886 (0.809–0.94)	0.812 (0.663–0.909)	0.880 (0.8–0.936)	0.930 (0.861–0.971)	0.944 (0.906–0.983)
LR	0.870 (0.81.53–0.91)	0.870 (0.786–0.92)	0.856 (0.77–0.917)	0.885 (0.804–0.941)	0.740 (0.542–0.864)	0.89 (0.822–0.934)	0.85 (0.779–0.90)	0.918 (0.870–0.966)

*EBM* explainable boosting machines, *LR* logistic regression, *PPV* positive predictive value, *NPV* negative predictive value.

**Figure 2:**
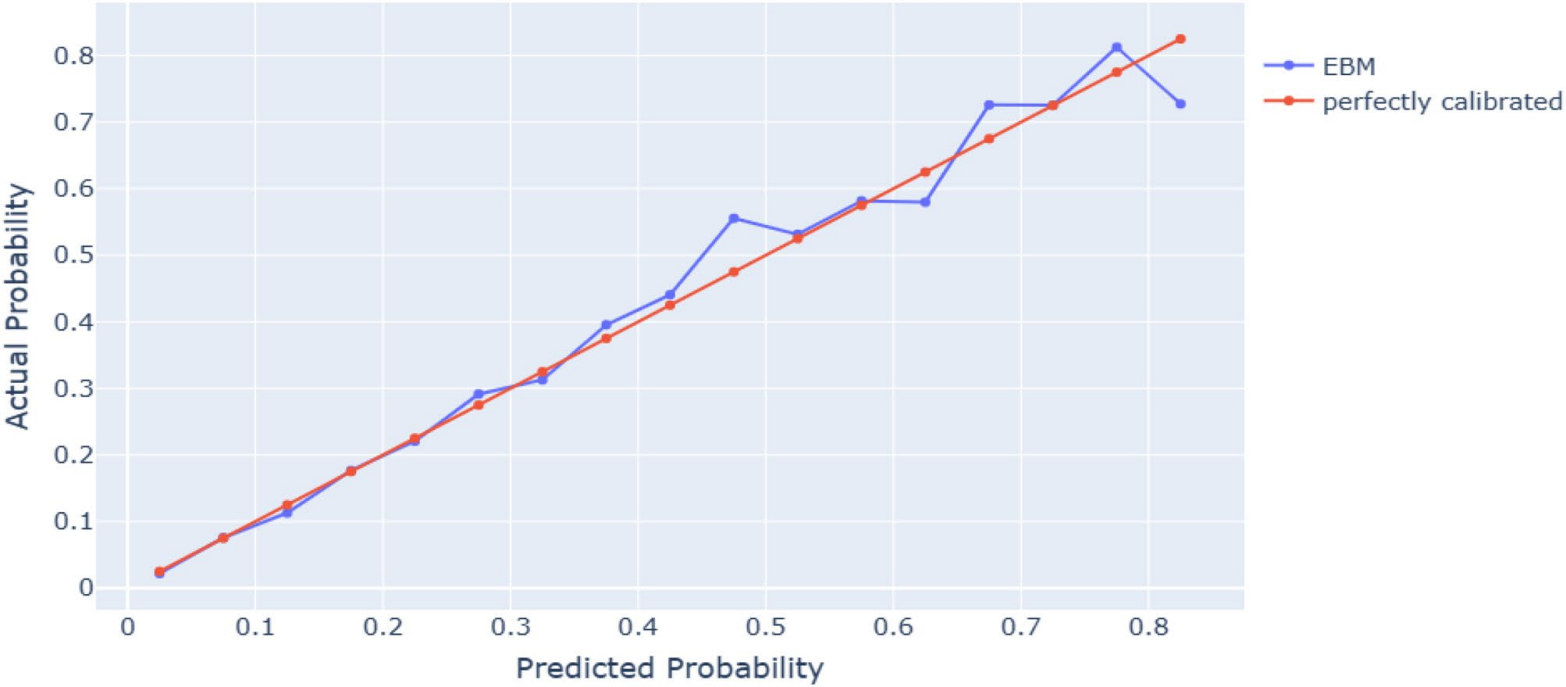
Calibration curve of the EBM-isotonic model.

As for important tasks such as AP prediction, the probabilities predicted from the models must reflect the true final probabilities, and for this purpose, we use the calibration plot based on isotonic regression. The calibration curve for the EBM model is presented in Fig. 2. The means of the calculated probabilities vs. the actual probabilities in each bin are plotted in scatter plots using the data, which is then separated into nearly equal frequency bins. The predicted probability may very well correspond to the actual probability in these bins as better-calibrated predictions are closer to the diagonal. Isotonic calibration was shown to have a good effect as a model calibration technique, and the EBM model demonstrated great agreement between the predicted and actual results.

### Global feature importance results

**Figure 3:**
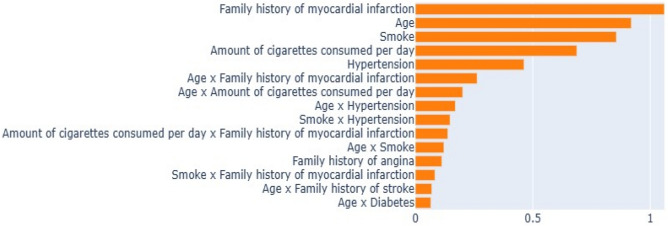
Global explanation of the EBM model.

The EBM algorithm is a generalized aggregation model based on the tree-based model. Due to additivity, the contribution of features can be graded and plotted to show the effect on individual prediction in both global and local directions. The general description of the EBM allows us to visualize the effect of each combination of parameters on the predicted results of the AP. Figure 3 summarizes the overall importance of each combination parameter, showing the importance of all combination factors. Family history of myocardial infarction, age, and smoking play a decisive role in AP. In addition, the EBM explanations were consistent with the results determined as a risk factor by considering the OR in the LR model in the prediction of AP (Fig. 3).

### Local explanation results

**Figure 4:**
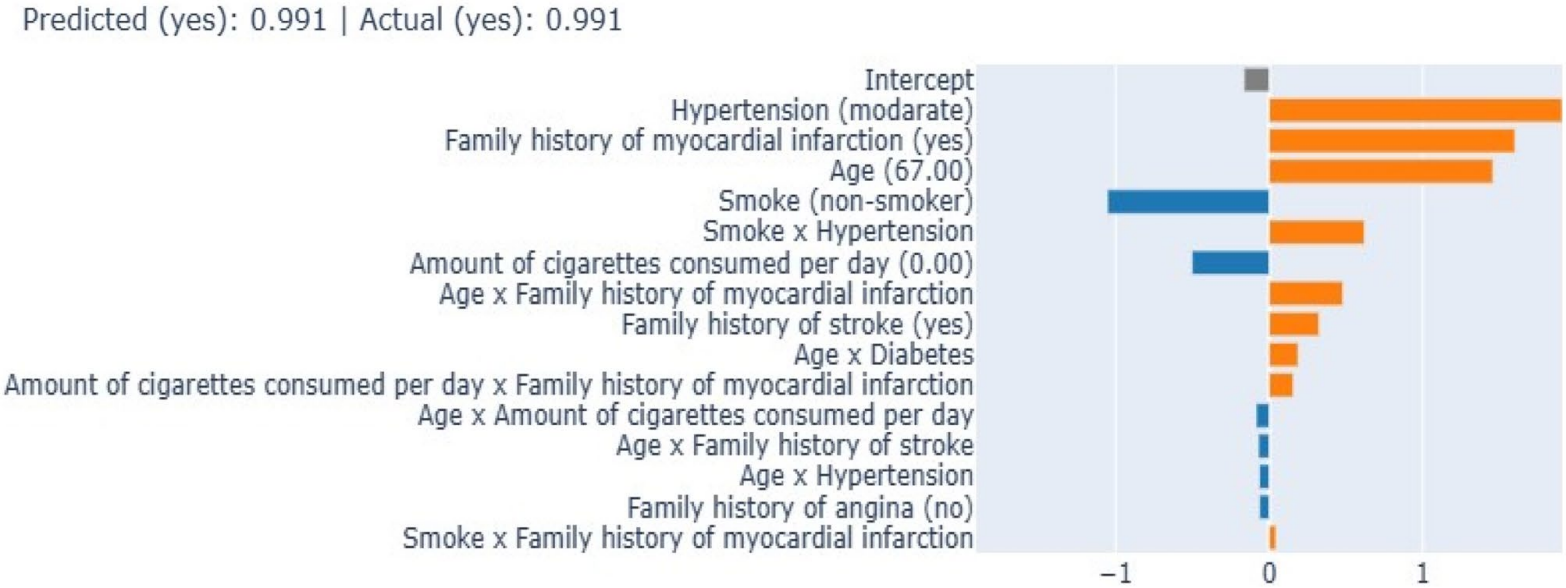
Local explanation of a true positive prediction result; orange represents the positive contribution to the AP prediction, and blue represents the negative contribution.

The EBM algorithm serves as a powerful tool in predictive modeling, particularly in its ability to provide granular insights into the contributions of individual variables for a single prediction. As illustrated in Fig. 4, the local annotation results for a typical individual Acute Pancreatitis (AP) prediction are presented. Remarkably, the algorithm predicted a risk of AP at 99.1%, which aligns precisely with the experimental value obtained through clinical evaluation. In dissecting the contribution of each variable to the predicted AP outcomes, several noteworthy observations can be made. Firstly, a non-smoking status was found to exert a negative impact on the predicted outcomes. This suggests that, within the context of this model, non-smoking serves as a protective factor against the development of AP. Conversely, variables such as moderate hypertension, a family history of myocardial infarction, and advanced age were identified as having a positive impact on the prediction decision. These variables, therefore, emerge as risk factors that elevate the likelihood of AP occurrence according to the model's calculations (Fig. 4).

## Discussion and conclusion

The overarching goal of the current research undertaking is to develop an effective and accurate predictive framework for AP, a cardiovascular condition characterized by chest pain or discomfort due to reduced blood flow to the heart muscle. Simultaneously, the study aims to unravel and analyze the intrinsic risk factors intricately linked to the manifestation of this ailment. To achieve these objectives, a multifaceted approach incorporating diverse ML methodologies will be harnessed. AP, a key indicator of underlying heart disease, necessitates timely diagnosis and management to avert potential complications. Anticipating the likelihood of its occurrence holds substantial clinical significance, enabling healthcare professionals to make informed decisions and proactively engage in preventive measures^4,5^. This research endeavors to harness the potential of ML algorithms to design predictive models capable of reliably estimating the probability of AP development in individuals.

In this clinical setting, we delve into a study that employs a comprehensive approach involving both multiple logistic regression analysis with backward feature selection and various tree-based machine-learning models—EBM, CatBoost, AdaBoost, XGBoost, and LightGBM—to predict the occurrence of AP and delineate its pivotal risk factors. The research findings shed light on the intricate relationships between independent variables and their impact on AP, offering critical insights for clinical practice and patient care. Furthermore, the significance of the effects is quantified through effect size results, which measure the magnitude or severity of each effect. Crucially, the validity of the model is established through rigorous statistical assessments. The Hosmer–Lemeshow test, a benchmark for model fit, yields a significant result (*p* > 0.05), signifying an acceptable and sufficient fit of the model to the data. The omnibus test bolsters this notion by revealing the overall significance of the model coefficients (*p* < 0.001), attesting to the collective importance of the variables in predicting AP. The results unearth pivotal risk factors that significantly influence the likelihood of AP occurrence. Notably, age demonstrates a direct association, as each one-unit increase escalates the risk by 1.125 times (OR = 1.125, 95% CI = [1.071–1.19], *p* < 0.001). Similarly, the mean amount of cigarettes consumed daily presents a noteworthy correlation, with a one-unit increase elevating the risk by 1.078 times (OR = 1.078, 95% CI = [1.031–1.133], *p* = 0.002). Furthermore, intriguing patterns emerge among various risk factors. Non-smoking status emerges as a protective factor, with the risk of AP plummeting by a substantial 7.04-fold in non-smokers compared to smokers (OR = 0.142, 95% CI = [0.045–0.407], *p* < 0.001). The influence of family history is also compelling, as having a family history of myocardial infarction remarkably elevates the risk by an astounding 11,226 times (OR = 11.226, 95% CI = [4.342–32.469], *p* < 0.001), marking the highest odds ratio observed. A family history of AP escalates the risk by 4.246 times (OR = 4.246, 95% CI = [1.139–17.891], *p* = 0.038). The study further underscores the impact of hypertension levels. Mild hypertension and moderate hypertension amplify the risk by 4.008-fold (OR = 4.008, 95% CI = [1.378–12.416], *p* = 0.013) and 9.38-fold (OR = 9.38, 95% CI = [1.685–64.42], *p* = 0.014), respectively. These findings illuminate the interplay of variables in AP prediction and offer crucial insights for clinical decision-making and patient management. This research enhances our understanding of AP etiology by harnessing advanced statistical techniques and ML models, facilitating tailored interventions, risk assessment, and improved patient outcomes.

The performance evaluation of the EBM model in predicting AP yields compelling insights into its predictive capabilities. The EBM model attains an accuracy and F1-score of 0.955 (95% CI: 0.926–0.984), reflecting its efficacy in classifying instances of AP correctly. This underscores the robustness of the EBM algorithm in capturing complex patterns and relationships within the dataset. In comparison to the other prediction models—CatBoost, AdaBoost, XGBoost, and LightGBM—it becomes evident that the EBM model outperforms its counterparts. Its superior performance indicates its ability to extract meaningful features and optimize predictive accuracy, further solidifying its potential as a powerful tool for AP prediction. An analysis of sensitivity, specificity, the Youden index, PPV, NPV, and AUC reveals the comprehensive nature of the EBM model's performance. EBMs are glass-box models often as accurate as state-of-the-art black-box models, e.g., neural networks, while remaining completely interpretable. Compared to other modern algorithms, CPAs are extremely compact and fast in prediction time. To understand how our proposed model is behaving, EBM offers two kinds of explanations: global and local, revealing that the presence of pairwise interactions between independent variables could provide good performance in predicting AP. The overall importance ranking (global explanation) of features was obtained by ordering their average absolute contribution in predicting the dependent variable AP. The local explanation of test subjects was also assessed as the ranking of the most important features in the single prediction, calculated as the logit of the probability (logarithm of the odds), where the logit of each feature is summed up for obtaining the final prediction. Figure 2 presents the overall feature importance in the AP classification using EBM. The Mean Absolute Value (MAS), on the x-axis, is used to calculate the overall ranking of the most important features contributing to the AP classification. It is observed that features such as family history, age, smoking, etc., are the most predictive features in AP classification. The local explanation of the classified AP using the EBM model for the gameplay dataset is shown in Fig. 3, where the MAS for both the true and predicted classes is 0.991, respectively, for the accurate classification of AP. In addition, most of the features contribute to the actual AP classification except non-smokers and consumption per day. A recent article similar to our work has used a Bi-LSTM model with an attention layer to predict AP from resting-state RR intervals and used data from the Sleep Heart Health Study database, which included 2,977 people followed for 15 years. The Bi-LSTM model has shown excellent predictive performance with accuracy = 0.913, AUC = 0.922, and precision = 0.913 in the testing set^15^. The EBM model in the current study outperformed the predictive performance of the Bi-LSTM model (0.955 vs. 0.913 in accuracy; 0.90 vs. 0.825 in PPV; 0.955 vs. 0.892 in F1-score)^15^.

Upon clinical examination, several significant risk factors emerged as reliable predictors for AP. Among these, smoking emerged as a prominent contributor, underscoring the detrimental impact of tobacco on cardiovascular health. Age also featured prominently, reflecting the cumulative effect of time on the cardiovascular system. The quantity of cigarettes smoked daily exhibited a dose-dependent relationship, reinforcing the role of tobacco intensity in angina development. Furthermore, a family history of myocardial infarction stood out as a hereditary factor, highlighting the genetic predisposition to cardiac ailments. Hypertension, a well-recognized risk factor, demonstrated its pivotal role in angina prediction, emphasizing the importance of blood pressure management. These findings collectively underscore the multi-faceted nature of angina pectoris prediction, where modifiable factors like smoking and hypertension intersect with non-modifiable elements such as age and family history^10,11^. Clinicians can leverage these insights to enhance risk assessment and develop tailored intervention strategies. A comprehensive approach that targets these risk factors could mitigate the likelihood of angina pectoris development and aid in promoting better cardiovascular health. The present study endeavors to leverage diverse ML methodologies to predict AP and unravel the associated risk factors. By amalgamating medical expertise, data science methodologies, and advanced predictive modeling techniques, this research reveals to enhance our understanding of AP, ultimately leading to improved patient care, timely interventions, and enhanced cardiovascular health outcomes.

This study established a prediction model for the occurrence of AP events through an explainable or interpretable approach based on EBM, and the developed model achieved good prediction performance. Detecting the occurrence of AP events using an EBM is vital for the healthcare sector. This predictive model can help clinicians monitor AP patients. An intelligent prediction of angina events could be achieved with the help of this study.

## Future works

### Generalizability and user interface

One of our primary objectives for future work is to test the robustness and applicability of our approach across various datasets. By doing so, we aim to ensure that our methodology is not limited to a specific type of data but is generalizable across different domains. Once the generalizability is confirmed, the next step would be to translate the algorithmic approach into a user-friendly interface. This interface will be specifically designed for medical professionals, including doctors and healthcare practitioners, to facilitate easier adoption and practical utility in clinical settings.

### Dataset construction

Another significant avenue for future research is the construction of a more comprehensive dataset. Our current dataset has limitations in terms of the range and depth of clinical and demographic features it covers. Therefore, we plan to include additional variables such as blood cholesterol levels, obesity indices, gender-specific data, and other relevant features. This enriched dataset will allow us to refine our models further and potentially uncover new insights into the problem at hand.

### Exploration of other boosting algorithms

In this study, we focused on some boosting algorithms such as EBM. EBMs can produce complex models, especially when the dataset is large or high-dimensional. As models become more complex, their decisions become harder to interpret and explain, making EBM impractical. In future research, hybrid models that combine the strengths of EBM with other interpretable models or techniques may be proposed. Combining different techniques can lead to more effective and interpretable models. Boosting algorithms, fundamentally, are ensemble techniques that combine the predictions from multiple machine learning algorithms to make more accurate predictions than any individual model. This approach is particularly powerful in scenarios where single models tend to underperform due to the complexity of the data or the subtlety of the patterns to be learned. While our current work has focused on specific boosting algorithms, we acknowledge that the field offers a plethora of other algorithms that have not been sufficiently explored. Some of these algorithms include LPBoost, TotalBoost, BrownBoost, MadaBoost, LogitBoost, and so on. LPBoost employs linear programming to optimize the margin between classes, aiming to find the best combination of weak classifiers^56^. TotalBoost is designed to maximize the minimum margin and is robust against noise in the data^57^. BrownBoost is particularly useful for dealing with noisy data and aims to avoid overfitting^58^. MadaBoost is a variant of AdaBoost that is designed to be more robust to noisy data and outliers^59^. Lastly, LogitBoost is designed to minimize logistic loss and is particularly useful for probabilistic classification. The algorithms we have mentioned, such as LPBoost and TotalBoost, may require significant computational resources, which could be a drawback in real-time applications. Additionally, algorithms like BrownBoost and MadaBoost, designed to handle noisy data, may not perform as well on clean, well-structured datasets.

## Data Availability

The public dataset used in this study can be requested from the corresponding author.
